# Factors associated with follow-up difficulty in longitudinal studies involving community-dwelling older adults

**DOI:** 10.1371/journal.pone.0237166

**Published:** 2020-08-03

**Authors:** Hisashi Kawai, Manami Ejiri, Harukazu Tsuruta, Yukie Masui, Yutaka Watanabe, Hirohiko Hirano, Yoshinori Fujiwara, Kazushige Ihara, Masashi Tanaka, Shuichi Obuchi

**Affiliations:** 1 Tokyo Metropolitan Institute of Gerontology, Tokyo, Japan; 2 Gerodontology, Department of Oral Health Science, Faculty of Dental Medicine, Hokkaido University, Sapporo, Japan; 3 Faculty of Medicine, Hirosaki University, Hirosaki city, Aomori, Japan; 4 National Institutes of Biomedical Innovation, Health and Nutrition, Tokyo, Japan; Pennington Biomedical Research Center, UNITED STATES

## Abstract

This study aims to clarify the factors associated with the gradual withdrawal from society in older adults. We defined the stages of follow-up difficulty based on four follow-up surveys on non-respondents of longitudinal mail surveys in community-dwelling older adults to examine the main factors associated with the stages of follow-up difficulty. We conducted a follow-up mail survey (FL1) with respondents of a baseline survey, and three more follow-up surveys with the non-respondents of each previous survey: simplified mail (FL2), postcard (FL3), and home visit surveys (FL4). The respondents of each follow-up survey were defined as a stage of follow-up difficulty; their characteristics concerning social participation and interaction at baseline in each stage were analyzed. The number of respondents in the FL1, FL2, FL3, and FL4 stages and non-respondents (NR) were as follows: 2,361; 462; 234; 84; and 101, respectively. Participation in hobby groups in FL2 and FL3, sports groups in FL4, and neighborhood association and social isolation in NR were significantly associated with the stage of follow-up difficulty. Based on these results, we conclude that the following factors are associated with each stage of follow-up difficulty: 1) a decline in instrumental activities of daily living in the FL2 and FL3 stages, 2) dislike for participating in physical activity such as sports in the FL4 stage, and 3) social isolation, not even belonging to a neighborhood association due to low social interaction in the NR group.

## 1. Introduction

Many studies have been conducted on the characteristics of those who drop out of a longitudinal study to assess the representativeness of participants in follow-up surveys [1–7]. These studies have reported that individuals who drop out of a longitudinal study often have characteristics such as poor health status, low socio-economic status—for example, poor education and economic status—and low social participation. They have also indicated that if baseline participants with these characteristics drop out of a longitudinal survey, it will have a significant impact on the findings of the study, implying that those who are likely to require assistance cannot be effectively assessed [6, 7].

However, it is possible to conduct follow-up surveys by using various methods—such as a simplified mail survey or a home visit survey—to examine the main findings of a longitudinal study concerning those who did not respond to the survey. The results suggest that there are people who participate in a survey without dropping out if researchers follow up carefully or if they change the survey method, such as presenting an easy-to-answer survey or a home visit survey. Therefore, we consider that the previous non-responders who responded to follow-up surveys of a longitudinal study would not have dropped out from the survey if they had received appropriate support from researchers and if researchers used various methods to improve the response rate. There is a possibility that respondents who did respond could be in somewhat better health and have higher socio-economic status than those who dropped out of the longitudinal study completely.

One factor of dropping out of a longitudinal study is low social participation [1]; dropout of a study may have similar nature to withdrawal from social activities. Thus, identifying the factors associated with respondents dropping out of a study—for example, if they do not receive appropriate support or if they find responding too difficult—can be useful for investigating the factors that restrict social participation in older adults. Indeed, social participation is reported to be associated with risk of functional disability in older adults [8], and social relationships and activities are important for prevention of frailty [9]. Recognizing these factors is crucial to prevent frailty and to maintain living function, thereby realizing a healthy life expectancy [10]. The International Classification of Functioning, Disability and Health defines living function as a combination of “body functions and structures,” “activities,” and “participation.” This means that not only physical and mental function but also daily life activities and social participation are essential to maintain living function [11].

Previous studies have identified various factors that are associated with social participation among older adults, including physical, psychological, social, and environmental factors [12–14]. Restricted social participation in older adults may be related to not only physical dysfunction, but also to the amount of social support that the older adults receive [14]. Therefore, restricted social participation in older adults may be associated with changes to their conditions—their physical condition as well as their psychological and social conditions—which can be improved with appropriate support. The characteristics of these conditions can be understood by analyzing the responses to the follow-up surveys conducted with non-respondents of a longitudinal study.

We conducted follow-up surveys with the non-responders of a longitudinal mail survey by means of a simplified mail survey, a postcard survey, and a home visit survey. We also identified participants who did not drop out from the survey because the survey had been simplified and was easier to answer. We then defined the stages of follow-up difficulty in a longitudinal study. To determine the baseline status of social participation and interaction—which are associated with the stages of follow-up difficulty—it is necessary to understand the characteristics of the participants whose social participation is gradually being restricted and to invent effective initiatives to improve social participation. To the best of our knowledge, no prior studies have been done on this subject. Therefore, we aimed to clarify the factors associated with the stages of follow-up difficulty in longitudinal surveys on community-dwelling older adults.

## 2. Methods

### 2.1 Participants

**Fig 1:**
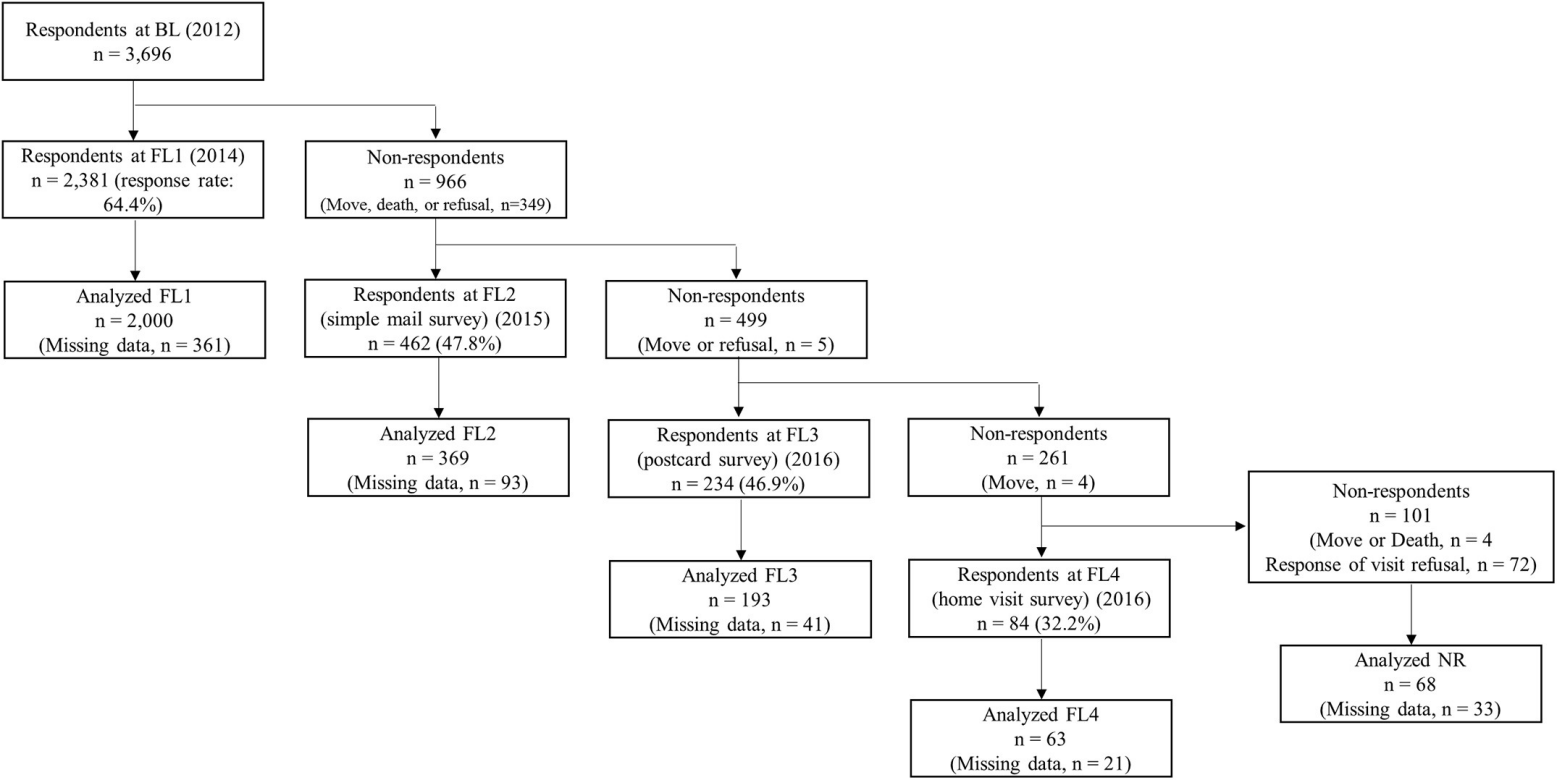
Flow diagram of study participants and the follow-up surveys. BL = Baseline survey, FL = Follow-up survey. Missing data: either social participation, social interaction, TMIG Index of Competence, self-rated health, or perceived financial status data missing at BL.

The participants in this study were the respondents of a mail survey for a cohort study that was conducted among community-dwelling older Japanese. The details of this cohort are also described in our previous study [15]. We conducted a mail survey that included all 7,015 residents who lived in nine areas in Itabashi-ku, Tokyo, Japan and who were aged between 65 and 85 [16]. We excluded institutionalized residents and participants of previous surveys conducted by our institute, and distributed a self-administered questionnaire with questions concerning lifestyle and health status. The study period was from August to October 2012 (baseline survey: BL). A follow-up mail survey was conducted from August to October 2014 with 3,696 BL respondents, of which 2,361 answered (follow-up 1: FL1). Next, follow-up surveys were conducted with non-respondents of each previous survey (FL1 to 4) (Fig 1).

For those who did not respond to FL1, a simplified mail survey with a reduced number of questionnaire items (from 24 to 10 items), was conducted from September to October 2015, and 462 people responded (follow-up 2: FL2). Concerning those who did not respond to FL2, a postcard survey with an even smaller number of questionnaire items (5 items) was conducted from February to April 2016, and 234 people responded (follow-up 3: FL3). Lastly, for those who did not respond to FL3, a home visit survey was conducted by examiners visiting the residents in June 2016, and 84 people responded (follow-up 4: FL4). The number of those who did not respond to any of the follow-up surveys were 101 (non-respondents: NR). Those who refused the offer of a home visit to conduct the survey in advance were not included in NR. The stages of follow-up difficulty were defined according to the follow-up surveys, with the lowest difficulty allocated to FL1 respondents and the highest difficulty allocated to FL4 respondents. The participants were assigned a code to classify them into four groups according to the stages of follow-up difficulty (i.e., FL1 = 0, FL2 = 1, FL3 = 2, FL4 = 3, and NR = 4).

Ethical approval for the study was granted by the ethics committee of the Tokyo Metropolitan Institute of Gerontology (Acceptance no. 61, 2013). The purpose of this study and the information privacy statement were provided in a briefing document that was included in the questionnaire or provided at the home visit, and all participants provided written informed consent to participate in this study.

### 2.2 Social participation and social interaction

We examined social participation of older adults in five activity groups: neighborhood associations, senior citizen clubs, hobby groups, sports groups, and volunteer groups [17]. Respondents were asked whether they were currently participating in any of these groups; their answers associated with each group were coded as “participation = 1” or “not participation = 0.”

Concerning social interaction, respondents were asked about the frequency of face-to-face contact and non-face-to-face contact (talking on the phone or communication via e-mail or letter) with non-resident family and friends. Respondents who answered that they had contact with relatives and friends “less than once a week” were defined as socially isolated; responses were coed as “isolation = 0” or “not isolation = 1” [18].

### 2.3 Covariates

In previous studies, age, sex, instrumental activities of daily living (IADL), self-rated health, and perceived financial status were indicated as factors associated with drop-out from a longitudinal study [1, 3–5, 19, 20]. For the current study, these factors were used as covariates for the analyses.

The question on self-rated health provided four choices: very healthy, healthy enough, not very healthy, and not healthy. IADL was assessed with a subscale of the TMIG Index of Competence that includes five questions on Instrumental Self-Maintenance [21]. The question on perceived financial status was assessed with five options: very comfortable, a little comfortable, neither comfortable nor hard, a little hard, very hard.

### 2.4 Statistical analysis

Participants with complete data for social participation, social interaction, and all covariates at BL were included in the statistical analyses. Differences concerning social participation, social interaction, and covariates between FL1, FL2, FL3, FL4, and NR at BL were examined by a chi-squared test for categorical variables, a one-way analysis of variance, and a post-hoc Bonferroni test for continuous variables. Linear trends among the stages of follow-up difficulty were assessed by the Jonckheere-Terpstra trend test or chi-squared test for trends. To examine the factors associated with the stage of follow-up difficulty from participants’ characteristics at BL, multinomial logistic regression analyses were conducted using the stage of follow-up difficulty as the dependent variable, and social participation and social interaction at BL as the explanatory variables. Two logistic regression models were calculated; one with the sex and age covariates adjusted (Model 1) and one with all covariates adjusted (Model 2).

Statistical analyses were performed using SPSS version 25 (IBM Japan, Ltd., Tokyo, Japan). Statistical significance was set at *p* < 0.05.

## 3. Results

Participants’ characteristics are shown in Table 1. The FL4 respondents were significantly older than the FL1 and FL2 respondents. The IADL scores for the FL2, FL3, and FL4 respondents were significantly lower than that of the FL1 respondents. There were no significant differences of proportion between men and women among the follow-up difficulty stages.

**Table 1:** Characteristics of participants at the baseline survey among the follow-up difficulty stages.

	FL1 (n = 2,000)		FL2 (n = 369)		FL3 (n = 193)		FL4 (n = 63)		NR (n = 68)		P for trend^a^	Significant Differences^b^
	Mean	SD	Mean	SD	Mean	SD	Mean	SD	Mean	SD		
Age (years)	72.5	5.3	72.3	5.5	72.7	5.7	74.6	5.9	72.7	5.6	0.727	FL4>FL1,FL2
IADL score	4.9	0.6	4.7	1.0	4.5	1.2	4.6	1.1	4.6	1.0	<.001***	FL1>FL2,FL3,FL4; FL2>F3
Sex	n	%	n	%	n	%	n	%	n	%	0.920	
Men	878	43.9	159	43.1	89	46.1	32	50.8	26	38.2		
Women	1122	56.1	210	56.9	104	53.9	31	49.2	42	61.8		
Social Participation												
Neighborhood associations	588	29.4	93	25.2	47	24.4	15	23.8	10	14.7	0.001**	
Senior citizen clubs	248	12.4	43	11.7	14	7.3	8	12.7	8	11.8	0.243	
Hobby groups	687	34.4	101	27.4	42	21.8	16	25.4	16	23.5	<.001***	FL1>FL3
Sports groups	473	23.6	73	19.8	31	16.1	3	4.8	11	16.2	<.001***	FL1>FL4, FL2>FL4
Volunteer groups	176	8.8	20	5.4	8	4.1	4	6.3	4	5.9	0.013*	
Social isolation												
Isolation	471	23.5	92	24.9	59	30.6	19	30.2	27	39.7	<.001***	NR>FL1
Self-rated Health											<.001***	
Very healthy	242	12.1	30	8.1	15	7.8	3	4.8	3	4.4		
Healthy enough	1396	69.8	245	66.4	119	61.7	37	58.7	41	60.3		
Not very healthy	288	14.4	72	19.5	46	23.8	15	23.8	17	25.0		FL3>FL1
Not healthy	74	3.7	22	6.0	13	6.7	8	12.7	7	10.3		FL4>FL1
Perceived financial status											<.001***	
Very comfortable	64	3.2	8	2.2	6	3.1	4	6.3	1	1.5		
A little comfortable	698	34.9	117	31.7	49	25.4	10	15.9	14	20.6		FL1>FL4
Neither comfortable nor hard	838	41.9	154	41.7	87	45.1	31	49.2	30	44.1		
A little hard	328	16.4	71	19.2	39	20.2	15	23.8	19	27.9		
Very hard	72	3.6	19	5.1	12	6.2	3	4.8	4	5.9		

SD = standard deviation; IADL = instrumental activities of daily living; FL = follow up; NR = non responders.

^a^Jonckheere-Terpstra trend test or Chi-square test for trend.

^b^Bonfferoni post-hoc test.

* *p* < .05;

** *p* < .01;

*** *p* < .001.

The percentage of participants that belonged to hobby groups were significantly higher in the FL1 group than in the FL3 group. The percentage of participants that took part in sports groups were significantly higher in the FL1 and FL2 groups than in the FL4 group. The percentage of respondents that were categorized as isolated was significantly higher in the NR group than in the FL1 group.

The percentage of respondents that rated themselves as “not very healthy” in the self-rated health question was significantly higher in the FL3 group than in the FL1 group. Additionally, the percentage of respondents that rated themselves as “not healthy” was significantly higher in the FL4 group than in the FL1 group. Lastly, the percentage of respondents that answered “a little comfortable” in the perceived financial status question was significantly higher in the FL1 group than in the FL3 group.

The multinomial logistic regression analysis in Model 1 with the adjusted sex and age covariates showed that non-participation in hobby groups was increased for the FL2 and FL3 respondents, non-participation in sports groups was increased for the FL4 respondents, and non-participation in neighborhood associations as well as isolation were increased in the NR group (Table 2). In Model 2—with all covariates adjusted—social participation and isolation were not significantly associated with the FL2 and FL3 respondents. However, a marginally significant association was shown between non-participation in hobby groups and the FL3 respondents, which is similar to the results from Model 1 (*p* = 0.091). Concerning the FL4 respondents and NR, a similar tendency as in Model 1 was observed.

**Table 2:** Age- and sex-adjusted and multivariate-adjusted odds ratios for the follow-up difficulty stages with social participation and isolation.

Model 1 (Age- and sex-adjusted)								
Follow-up Difficulty (reference: FL1)								
	FL2		FL3		FL4		NR	
	OR	(95% CI)	OR	(95% CI)	OR	(95% CI)	OR	(95% CI)
Social participation (reference: participation of each social group)								
Neighborhood associations	1.16	(0.89–1.52)	1.05	(0.73–1.50)	1.25	(0.66–2.35)	**2.29**	(1.12–4.70)
Senior citizen clubs	0.90	(0.62–1.31)	1.56	(0.87–2.81)	0.93	(0.41–2.09)	0.68	(0.30–1.52)
Hobby groups	**1.30**	(1.00–1.69)	**1.57**	(1.08–2.28)	1.14	(0.62–2.11)	1.38	(0.75–2.55)
Sports groups	1.16	(0.87–1.55)	1.33	(0.88–2.01)	**5.29**	(1.63–17.23)	1.27	(0.64–2.52)
Volunteer groups	1.49	(0.91–2.44)	1.69	(0.81–3.56)	0.93	(0.32–2.73)	0.98	(0.34–2.83)
Social isolation (reference: not isolation)								
Isolation	0.98	(0.75–1.29)	1.19	(0.85–1.67)	1.07	(0.60–1.91)	**2.01**	(1.18–3.41)
Model 2 (Multivariate-adjusted^a^)								
Follow-up Difficulty (reference: FL1)								
	FL2		FL3		FL4		NR	
	OR	(95% CI)	OR	(95% CI)	OR	(95% CI)	OR	(95% CI)
Social participation (reference: participation of each social group)								
Neighborhood associations	1.12	(0.85–1.47)	0.96	(0.67–1.38)	1.16	(0.61–2.20)	**2.11**	(1.03–4.35)
Senior citizen clubs	0.91	(0.63–1.32)	1.61	(0.89–2.92)	1.00	(0.44–2.26)	0.71	(0.32–1.60)
Hobby groups	1.22	(0.93–1.59)	1.39	(0.95–2.04)	1.00	(0.54–1.87)	1.20	(0.64–2.24)
Sports groups	1.08	(0.80–1.44)	1.17	(0.77–1.79)	**4.55**	(1.39–14.91)	1.06	(0.53–2.12)
Volunteer groups	1.44	(0.88–2.36)	1.66	(0.78–3.51)	0.90	(0.30–2.68)	0.89	(0.30–2.60)
Social isolation (reference: not isolation)								
Isolation	0.93	(0.71–1.22)	1.09	(0.77–1.54)	0.94	(0.53–1.70)	**1.79**	(1.04–3.06)

CI = confidence interval; OR = odds ratio; FL = follow up; NR = non responders.

Bold numbers are statistically significant.

^a^Adjusted for age, sex, instrumental activities of daily living, self-rated health, and perceived financial status.

## 4. Discussion

To clarify the factors that contribute toward older adults’ gradual withdrawal from society, this study defined stages of follow-up difficulty in a longitudinal study and examined the characteristics of individuals at each stage of follow-up difficulty. As there are no existing studies that defined follow-up difficulty for non-respondents of a longitudinal survey, the present study is novel and offers a valuable theoretical contribution. Many previous studies have suggested that respondents’ tendency to drop out from a longitudinal study may be affected by poor health and socio-economic status at the baseline survey [1, 3–5, 19, 20]. Therefore, to compare the findings obtained in this study with those of previous studies, we first examined whether the FL1 respondents—those who did not drop out from the longitudinal survey in this study—had similar results to those of previous studies. The FL1 respondents showed statistically significant differences in age, IADL score, participation in hobby and sports groups, self-rated health, and perceived financial status compared to the other stages of follow-up difficulty. This suggests that better health condition and socio-economic status as well as higher physical function and social activity levels were characteristics of participants who did not drop out.

These results are concordant with several previous studies that examined attrition in longitudinal studies [1, 3–5, 20]. Therefore, although this study was conducted in one cohort of community-dwelling older adults in Japan, the results showed a common tendency across several previous studies including different countries, suggesting some universal basic characteristics associated with older adults who drop out of longitudinal surveys.

Follow-up surveys—which were gradually made easier to answer—were conducted with those who dropped out of this study’s longitudinal survey, and we investigated the response rate of each stage. It should be noted that such gradually changing follow-up surveys have not been conducted in previous studies, and no previous studies have focused on follow-up surveys from this perspective. The response rate of each follow-up survey was 47.8% (462/966) for the simplified mail survey (FL2), 46.9% (234/499) for the postcard survey (FL3), and 32.2% (84/261) for the home visit survey (FL4). This indicates that even if people dropped out from the survey once, approximately 30–50% of these individuals responded to the follow-up surveys that were altered to make them easier to answer. These results can be utilized to conduct follow-up surveys with non-responders of a longitudinal mail survey by changing the survey methods.

Contrary to our assumption that answering the follow-up questions of the surveys would be progressively easier when changing the survey method by implementing simplified mail, postcard, and home visit surveys, the response rate gradually declined. This indicates that the more difficult the follow-up is, the more problematic it is to obtain responses, even if the surveys themselves become easy to answer. Therefore, to explore the reasons for this decline, we examined the characteristics associated with the stage of follow-up difficulty.

It was observed that IADL gradually decreased along with the stage of follow-up difficulty. Although the results concerning some social activities were not consistent, it can be suggested that the participation rate in activities would also decrease along with follow-up difficulty. The results further showed that the percentage of isolation increased with the increase in follow-up difficulty. A similar tendency was observed concerning health and economic status. Therefore, the stage of follow-up difficulty defined in this study can be regarded to reflect the decline in physical function and social activity that are related to restricted social participation.

Next, we examined the baseline factors that determined the stage of follow-up difficulty. The factors involved in each stage were participation in hobby groups for the FL2 and FL3 respondents, participation in sports groups for the FL4 respondents, and participation in neighborhood associations and isolation for NR. Similar results were obtained concerning the FL4 and NR groups after covariate adjustment. These results were considered to be more robust than those of FL2 and FL3. A relatively small percentage of people (4.8%) participated in sports groups in FL4, which could indicate that many people who dislike participating in sports were included in this stage. Physical activity may also be low in this stage, making it difficult for respondents to answer even the postcard survey because of poor physical function. This may be the reason they responded only to the home visit survey. Moreover, a decline in cognitive function makes it difficult to respond to mail. Therefore, it is possible that the group who could not respond to the postcard survey and responded to the home visit survey instead may include persons with cognitive decline. Several studies have reported cognitive function as a factor of attrition in longitudinal studies [1].

In the NR group, there were many people with low social interaction, which contributes greatly to isolation. The participation rate for even local activities, such as neighborhood association, was low. This means that the NR group included many people with low social interaction and with poor social participation.

On the other hand, social participation did not prove to be a factor in the FL2 and FL3 groups after adjusting for covariates. It was, in fact, IADL that proved to be the factor more significantly associated with these stages. This suggests that the decline of daily living function made it difficult for people to respond in these stages. As Nemoto et al. [13] reported, social activity can be restricted by IADL disability, and the decline in baseline IADL may restrict future social participation in persons of those stages.

Based on these results, we conclude that the factors associated with each stage of follow-up difficulty are as follows: 1) a decline in IADL in the FL2 and FL3 stages, 2) dislike for participation in physical activity such as sports in the FL4 stage, and 3) social isolation, not even belonging to a neighborhood association, owing to low social interaction in the NR group. Older adults to which these factors apply are at risk of restricted social participation in the future. The results of this study also showed that the appropriate support needed to enable older adults to respond to longitudinal surveys differed among the stages of follow-up difficulty. The findings obtained in this study will be useful to prevent not only drop-out from surveys in a longitudinal study but also gradual reduction in social participation among older adults.

This study, however, has some limitations. Although the characteristics at the baseline of each stage of follow-up difficulty were examined, the characteristics at each follow-up survey were not compared with each other. However, we did determine that health outcomes—such as self-rated health and incidence of requirement of long-term care—differ for each of the follow-up surveys and between the stages of follow-up difficulty. These results will be reported in a future study. Another limitation of this study is that factors concerning non-respondents, such as moving away or death, were accurately examined using resident cards only for the FL2 survey. As for the other follow-up surveys, we identified the moving away or death factors only by information from participants or their family members. Therefore, non-responders for the FL3 group and later groups may include more moves and deaths. However, the surveys after the FL3 survey were conducted within one year, and the impact on the results obtained in this study would be small. Since this study was a longitudinal mail survey, it was not possible to assess details concerning educational status, work, and cognitive function, which have been indicated by prior studies to be associated with increased rates of attrition. In future studies, it is necessary to determine whether these factors are associated with follow-up difficulty.

## 5. Conclusions

This study identified the stages of follow-up difficulty based on four follow-up surveys with non-respondents of longitudinal mail surveys in community-dwelling older adults, thereby examining the factors associated with withdrawal from society in older adults. The results indicated that the most prominent factors in each stage associated with withdrawal from society were a decline in instrumental activities of daily living, dislike for participating in physical activity such as sports, and social isolation. These findings would be useful to address the gradual withdrawal from society in older adults.
